# CARP-1 Functional Mimetics: A Novel Class of Small Molecule Inhibitors of Medulloblastoma Cell Growth

**DOI:** 10.1371/journal.pone.0066733

**Published:** 2013-06-24

**Authors:** Abdelkader E. Ashour, Shazia Jamal, Vino T. Cheryan, Magesh Muthu, Khairy M. A. Zoheir, Ahmed M. Alafeefy, Adel R. Abd-Allah, Edi Levi, Adi L. Tarca, Lisa A. Polin, Arun K. Rishi

**Affiliations:** 1 John D. Dingell Veterans Affairs Medical Center, Detroit, Michigan, United States of America; 2 Karmanos Cancer Institute, Wayne State University, Detroit, Michigan, United States of America; 3 Department of Oncology, Wayne State University, Detroit, Michigan, United States of America; 4 Department of Computer Science, Wayne State University, Detroit, Michigan, United States of America; 5 Department of Pharmacology and toxicology, College of Pharmacy, King Saud University, Riyadh, Kingdom of Saudi Arabia; 6 Department of Pharmaceutical Chemistry, College of Pharmacy, Salman Bin Abdulaziz University, Alkharj, Kingdom of Saudi Arabia; 7 Cell Biology Department, National Research Center, Dokki, Cairo, Egypt; UCSF/VA Medical Center, United States of America

## Abstract

Medulloblastomas (MBs) constitute an aggressive class of intracranial pediatric tumors. Current multimodality treatments for MBs include surgery, ionizing radiation, and chemotherapy. Toxic side effects of therapies coupled with high incidence of recurrence and the metastatic spread warrant development of more effective, less toxic therapies for this disease. CARP-1/CCAR1 is a peri-nuclear phospho-protein that is a co-activator of the cell cycle regulatory anaphase promoting complex/cyclosome (APC/C) E3 ligase. CARP-1 functional mimetics (CFMs) are a novel class of small molecule compounds that interfere with CARP-1 binding with APC/C subunit APC-2, and suppress growth of a variety of cancer cells in part by promoting apoptosis. Here we investigated MB growth inhibitory potential of the CFMs and found that CFM-4 inhibits growth of MB cells in part by inducing CARP-1 expression, promoting PARP cleavage, activating pro-apoptotic stress-activated protein kinases (SAPK) p38 and JNK, and apoptosis. Gene-array-based analysis of the CFM-4-treated Daoy MB cells indicated down-regulation of a number of key cell growth and metastasis-promoting genes including cell motility regulating small GTP binding protein p21Rac1, and extracellular matrix metallopeptidase (MMP)-10. Moreover, CFM-4 treatment stimulated expression of a number of molecules such as neurotrophin (NTF)3, and NF-κB signaling inhibitors ABIN1 and 2 proteins. Overexpression of NTF3 resulted in reduced MB cell viability while knock-down of NTF3 interfered with CFM-4-dependent loss of viability. CFMs also attenuated biological properties of the MB cells by blocking their abilities to migrate, form colonies in suspension, and invade through the matrix-coated membranes. Together our data support anti-MB properties of CFM-4, and provide a proof-of-concept basis for further development of CFMs as potential anti-cancer agents for MBs.

## Introduction

Medulloblastoma is a common childhood brain cancer. It is a highly malignant tumor type with poor overall prognosis [1]. Current treatment options include a combination of surgery, radiation and chemotherapy. The disease however remains incurable in about a third of the patients, and the therapy-associated severe neurological toxic side effects often result in significant morbidity [2].

Although it is well known that most MBs originate from the distinct germinal zones of the cerebellar cortex [3], [4], the transforming events that initiate and/or sustain these cancers are yet to be elucidated. Differential expression of some antigens and receptors such as neurotrophin receptor p75^NTR^/TrkC is often noted in common variants of MB and serves as a marker of favorable outcome [5], [6]. Moreover, recent gene expression profiling studies have defined four MB subgroups that include sonic hedgehog subgroup, the WNT subgroup, and subgroups 3 and 4 [7]. Whole genome sequencing of a number of primary medulloblastoma tumors representing all the four subgroups further revealed that mutations in different epigenetic modifiers may distinguish MB subgroups 3 and 4, thus presenting potential for targeting of subgroup-specific alterations for therapeutic benefit [8].

Emerging evidence indicates that although majority of MBs harbor wild-type p53 [9], the tumor suppressor function of p53 is often impacted by the associated oncogenes such as MDM2 and/or WIP1/PPMID 10,11. In this context, recent, proof-of-principle studies have revealed that tumor suppressor functions of p53 can be restored in MBs. The micro-RNA miR-34a was found to sensitize MB cells to chemotherapy in part through its modulation of p53 [12], while a pharmacological inhibitor of MDM2 (nutlin-3) re-activated p53 function and suppressed growth of MB cells in vitro and in vivo [11]. Another recent study explored utility of modified measles virus for treatment of MBs in vitro and in intracerebral murine xenograft model [13]. Thus although significant advances have been made in understanding the biology of MBs, the current treatment modalities remain insufficient to effectively treat and manage this disease, and therefore, warrant development of new anti-MB treatment strategies.

We previously identified and characterized a peri-nuclear phospho-protein, termed CARP-1/CCAR1 [14], [15]. CARP-1 is a co-activator of the cell cycle regulatory anaphase promoting complex/Cyclosome (APC/C) E3 ligase [16] and the p53 [17], and is a key transducer of cell growth as well as chemotherapy (adriamycin, etoposide, or iressa)-dependent inhibitory signaling [14], [15], [17]. The APC/C co-activator function of CARP-1 was recently exploited to identify a number of small molecule inhibitors (SMIs) of CARP-1 binding with APC-2 subunit of APC/C. We have previously shown that these compounds, termed CARP-1 Functional Mimetics (CFMs), suppress growth of a variety of cancer cells in part by stimulating apoptosis [16]. Here we investigated MB growth inhibition by CFMs and the underlying molecular mechanisms. CFMs stimulated pleiotropic anti-MB cell growth signaling that included activation of apoptosis and attenuation of cell growth and survival. Last but not least, CFMs also interfered with biological properties of colony formation, invasion and migration of the MB cells. Thus, our proof-of-concept studies underscore MB inhibitory properties of CFMs that could facilitate development of CFMs or their derivatives/analogs as potential anti-MB modalities.

## Methods

### Cells and Reagents

The human medulloblastoma Daoy and UW-228-1 cells were obtained from Dr. Ronaldo DelMaestro (Brain Tumor Research Center, Montreal Research Institute and Hospital, Montreal, Canada) and were maintained essentially as described before [18]. All the cells were routinely cultured in DMEM supplemented with 10% fetal bovine serum (FBS), 100 units/ml of penicillin, and 100 µg/ml of streptomycin. Cells were maintained at 37°C and 5% CO_2_ and were passaged weekly. CFM-1, 4, and 5 were obtained from ChemDiv and/or ChemBridge, San Diego, CA, and dissolved in dimethyl sulfoxide (DMSO; Sigma; St. Louis, MO) at a stock concentration of 50 mM, aliquoted and stored at –20°C, and appropriate working concentrations were prepared with the cell culture medium immediately before the experiments. Clinical grade Adriamycin was from the Harper Hospital Pharmacy, Wayne State University, Detroit, MI. Purified anti-NT3 (neurotrophin 3) mouse monoclonal antibody, clone 5A2, and human NTF3 expression plasmid pCMV6-XL5-NTF3 variant 1 (catalog # sc316918) were obtained from Origene Technologies, Inc., Rockville, MD. Anti-Trk (C-15) rabbit polyclonal, anti-ABIN2 mouse monoclonal, and anti-APC-2 antibodies were purchased from Santa Cruz Biotechnology, Inc., CA. In addition, anti-TrkC mouse monoclonal (62CT51.7.7) and rabbit monoclonal (C44H5) antibodies were purchased from LifeSpan Biosciences, Inc., Seattle, WA and Cell signaling Technology, Beverly, MA, respectively. Anti-cyclin B1 and anti phospho-JNK (Threonine183/Tyrosine 185) G9 mouse monoclonal antibodies, anti-JNK (56G8) rabbit monoclonal antibody, and rabbit polyclonal antibodies for PARP, phospho and total p38 SAPK, ABIN1, IκBα, and IκBβ proteins were obtained from Cell Signaling Technology (Beverly, MA). Anti-p21 Rac1 mouse monoclonal antibody was purchased from BD Biosciences, San Jose, CA. Generation and characterization of the anti-CARP-1/CCAR1 rabbit polyclonal antibodies have been described before [14]. Enhanced Chemiluminescence Reagent was purchased from Amersham Biosciences (Piscataway, NJ) and the Protein Assay Kit was purchased from Bio-Rad Laboratories (Hercules, CA), while Anti β-Actin mouse monoclonal antibody, and chemicals including 3-4, 5-dimethyltiazol-2-yl-2.5-diphenyl-tetrazolium bromide (MTT), cremophor were obtained from Sigma-Aldrich (St. Louis, MO). Purified, lyophilized recombinant human pro-neurotrophin 3 and mature neurotrophin 3 proteins were obtained from BioVision Inc., Milpitas, CA and Cell Signaling, Beverly, MA, respectively, and resuspended in sterile PBS to obtain a 100 µg/ml stock of the respective proteins. The reconstituted protein stocks were aliquoted and stored at −20°C, and appropriate working concentrations were prepared immediately before the experiments. The ON-Target plus SiRNAs for knock-down of NTF3 and DharmaFECT transfection reagent for si-RNA transfections were purchased from Dharmacon Inc., Thermo Fisher Scientific (Lafayette, CO). The lipid-based transfection reagent Lipofectin 2000 for transfection of plasmid DNAs was purchased from Life Technologies, Grand Island, NY.

### MTT Assay, Caspase Activity Measurement and Western Blot (WB)

MB cells (5×10^3^) were seeded in a 96-well culture plate and subsequently treated with respective CFM at different concentrations for noted times. Control cells were treated with 0.1% DMSO in culture medium. After indicated periods of treatment, the cells were incubated with 1 mg/ml of MTT reagent at 37°C for 4 hours and then MTT was removed and the purple formazan crystals formed at the bottom of the wells were dissolved using 100 µl of DMSO, followed by colorimetric analysis using a multilabel plate reader at 570 nm (Victor^3^; PerkinElmer, Wellesley, MA).

Activation of caspases was measured by utilizing the ApoAlert Caspase profiling plate (Clontech) following the manufacturer suggested methods. Cell lysates derived from vehicle (DMSO) or CFM-treated cells were added to the chambers containing immobilized fluorogenic caspase-2, -8, -9, and -3 substrates. The fluroscence signal released from the activated caspase-mediated cleavage of the respective substrate was detected by a plate reader at the excitation and emission wavelengths of 380 and 460 nm, respectively.

For WB analyses, cells were harvested and lysed in RIPA buffer (50 mM Tris-HCI, pH 8.0, 150 mM sodium chloride, 1.0% NP-40, 0.5% sodium deoxycholate, 0.1% sodium dodecyl sulfate (SDS), and 0.1% of protease inhibitor cocktail) for 20 min at 4°C. The lysates were centrifuged at 14,000 rpm at 4°C for 15 min to remove debris. Protein concentrations of whole cell lysates were determined using the Protein Assay Kit. Supernatant proteins, 50 µg from each sample, were separated by SDS-10% polyacrylamide gel electrophoresis (SDS-PAGE) and transferred to polyvinylidene difluoride (PVDF) membrane (Bio-rad, Hercules, CA) by standard procedures. The membranes were hybridized with primary antibodies followed by incubation with appropriate secondary antibodies. The antibody-bound proteins were visualized by treatment with the chemiluminescence detection reagent (Amersham Biosciences) according to manufacturer’s instructions, followed by exposure to X-ray film (Kodak X-Omat). The same membranes were re-probed with the anti-β actin antibody, which was used as an internal control for protein loading.

### Isolation of RNA and Microarray Analysis

Total RNA was extracted from untreated or CFM-4-treated Daoy MB cells. The MB cells were treated with 2 µM, 6 µM and 8 µM dose of CFM-4 for 12 h in serum-free medium. At the end of treatments, the untreated and treated cells were harvested and total RNA were isolated, and purified using the RNeasy Mini kit and RNase-free DNase Set (Qiagen, Valencia, CA) according to the manufacturer’s protocols. CFM-4-dependent changes in gene expression in MB cells were performed at the Genomic Core Facility, Karmanos Cancer Institute utilizing Illumina BeadChip® Arrays essentially according to manufacturer’s instruction (Illumina Inc., San Diego, CA). Briefly, 0.5 µg total RNA was biotin-labeled and hybridized with BeadChips. The signal was detected with streptovadin-Cy3 according to manufacturer’s instruction (Illumina). The imaging of the BeadChips was conducted using a Bead Array Reader in conjunction with Bead Studio software (Illumina). Normalization of the data was carried out using a quantile based approach which transforms the raw data so that the resulting normalized expression values of each sample have the same distribution [19], [20]. Differential gene expression between the treated and untreated groups was tested using a moderated *t*-test. Genes were selected as significant using a cutoff of 0.1 on the nominal p-values and a cutoff of 1.5-fold change in expression between groups.

### NTF3 Knock-down Experiments

Daoy MB cells were transfected with scrambled or NTF3 SiRNAs utilizing Dharmafect reagent essentially following the manufacturer suggested protocols. After 96 h incubation with the SiRNAs, the cells were either lysed, and protein extracts analyzed by WB for expression of pro-NTF3, or cells were treated with various doses of CFM-4 and their viabilities were determined by the MTT assay as detailed above. In addition, a plasmid encoding variant 1 of the human pro-NTF3 (cat. # SC316918) was obtained from Origene Technologies, Rockville, MD. UW-228-1 MB cells were transfected with the vector or the pro-NTF3 expression plasmid. The levels of pro-NTF3 in the transfected cells and the viabilities of the transfected cells that were either untreated or treated with CFM-4 or CFM-5 were determined by WB and MTT assays, respectively. For stable NTF3 knockdown, we first PCR amplified pro-NTF3 cDNA from positions 191-698 (NM_001102654.1) and subcloned this PCR amplified cDNA in an antisense orientation to obtain a recombinant plasmid (pcDNA3/NTF3-AS Clone1). Daoy MB cells were then transfected with pcDNA3 vector or pcDNA3/NTF3-AS clone 1 plasmids, and the transfected cells were grown in the continuous presence of 200 µg/ml neomycin (G418) as described before [21]. Multiple, stable, neomycin-resistant sublines were isolated followed by determination of pro-NTF3 levels by WB essentially following the previously published methods [14], [16]. The stable sublines expressing vector or the reduced levels of pro-NTF3 were then treated with CFM-4 (4 µM) and CFM-5 (10 µM) for various time periods, and their viabilities were determined by the MTT assays as above.

### Cell Migration, Invasion, and Clonogenic Assays

The effects of CFMs on migration of MB cells were measured by the “scratch” assay. Cells were grown in a 6-well plate (∼10,000 cells/well), and a scratch was created in the cell monolayer using sterile pipette tip. The cells were then allowed to grow in the absence (Control) or presence of 10 µM dose of each of the CFMs for a period of 72–96 hours. Images were captured at the beginning and at regular intervals during cell migration to close the scratch, and the images were compared to quantify the migration rate of the cells essentially as described before [22]. The cells were photographed under different magnifications utilizing Zeiss microscope with attached 35 mm camera for recording the photomicrographs.

#### Clonogenic assay

Cells were sandwiched between 0.6% and 0.3% agarose in DMEM medium containing 5% FBS in a six-well chamber (500 cells/chamber), and treated with buffer (Control), or respective CFM (10 µM) for 9 days at 37°C humidified CO_2_ incubator. The colonies from multiple random fields were counted, compared to control and photographed essentially as above.

#### Invasion assay

Basement membrane is a thin extracellular matrix (ECM) that underlies epithelia and endothelia and separates epithelial cancer cells from the stroma. Tumor cells produce proteases that degrade ECM to cross the basement membrane to invade stroma and establish distant metastases. The in vitro assay using Matrigel is the most reliable, reproducible, and representative of in vivo invasion. In this Boyden Chamber assay, cancer cells are placed in the upper chamber that is separated from the lower chamber by a porous membrane coated with Matrigel [23]. A cell invasion Boyden chamber assay kit (Chemicon International, CA) was utilized to measure invasion properties of the cancer cells in the absence or presence of CFMs. Briefly, pre-warmed serum free medium (300 µl) was used to hydrate the ECM layer of each chamber for 15–30 minutes at room temperature. Approximately 2–2.5×10^5^ MB cells were seeded in the upper chamber in a serum-free medium without or with 10 µM dose of the respective CFM. The lower chamber was supplied with full medium containing 10% FBS that served as chemo-attractant to stimulate migration. After an interval, tumor cells present on the lower side of the membrane in the lower chamber were stained, and photographed as above. In addition, the stained cells from the lower side of membrane of some wells were dissociated, lysed in a buffer, followed by quantitation using a fluorescence plate reader with 480/520 nm filter set. The measurements were then plotted as bars in histogram.

### Immunocytochemical Analysis

Apoptosis in MB cells was determined by TUNEL assay using in situ cell Death Detection kit from Roche Applied Science (Indianapolis, IN) according to the manufacturer’s instruction. Approximately 5×10^3^ cells were seeded in each well of an 8-chamber slide (LabTek, CA), and allowed to grow overnight at 37°C incubator. The cells were then either untreated or treated with respective CFM, and processed for staining essentially following our previously described procedures [24]. The cells on the slides were then stained for presence of CARP-1, phospho-p38, TrkC, and NTF3 by utilizing respective antibodies, and were then photographed under different magnifications using Zeiss microscope with a 35 mm camera attached for recording the photomicrographs. H&E counter-staining of the cells was performed following our previously described methods [24].

### Detection of MMP Expression in Human MB Cells

Daoy cells were separately treated with CFM-4 or CFM-5, followed by their homogenization in RIPA buffer (500 µl of lysis buffer per 1×10^6^ cells). The cell lysates were centrifuged at 10,000×g for 5 min, and the protein concentration in the supernatant of the respective lysate was determined by using Bicinchoninic acid assay. The lysates were stored at −80°C until further use. MMP activation in each lysate was measured using the Quantibody reverse phase human MMP array kit according to manufacturer’s instructions (RayBiotech, Norcross, GA). Fluorescence images were detected using a GenePix 4100A Scanner, and data was analyzed using the QAH-MMP-1 GAL software based on the instruction provided by the array manufacturer.

### Statistical Analysis

Where appropriate, statistical analysis was performed using unpaired Student’s t-test. A p value less than 0.05 between treatment groups is considered significantly different.

## Results

### CFMs Inhibit MB Cell Proliferation in Part by Stimulating Apoptosis

**Figure 1:**
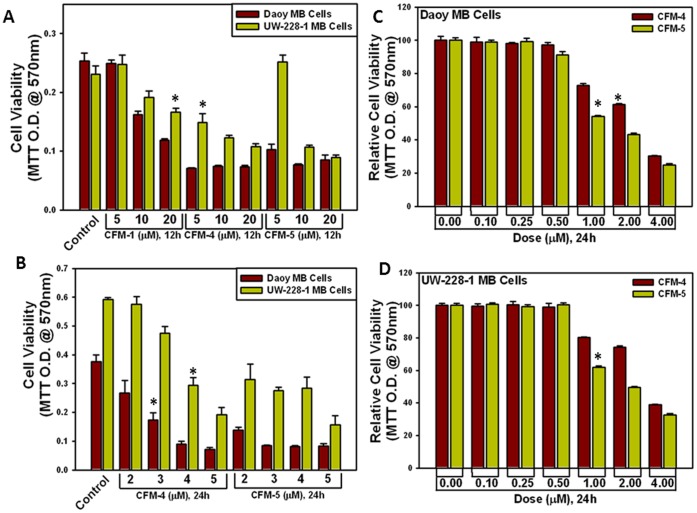
CFMs reduce viabilities of the human MB cells. Cells were treated with vehicle (Control) or indicated doses of CFMs for 12 h (A) or 24 h (B). In panel C and D, the cells were treated with respective CFM for noted dose and time. Determination of viable/live cells was carried out by MTT assay. The data in the histograms represent means of three independent experiments; bars, S.E. ∗, p = <0.05 relative to Control (A, B) or 0.00 (C, D).

**Figure 2:**
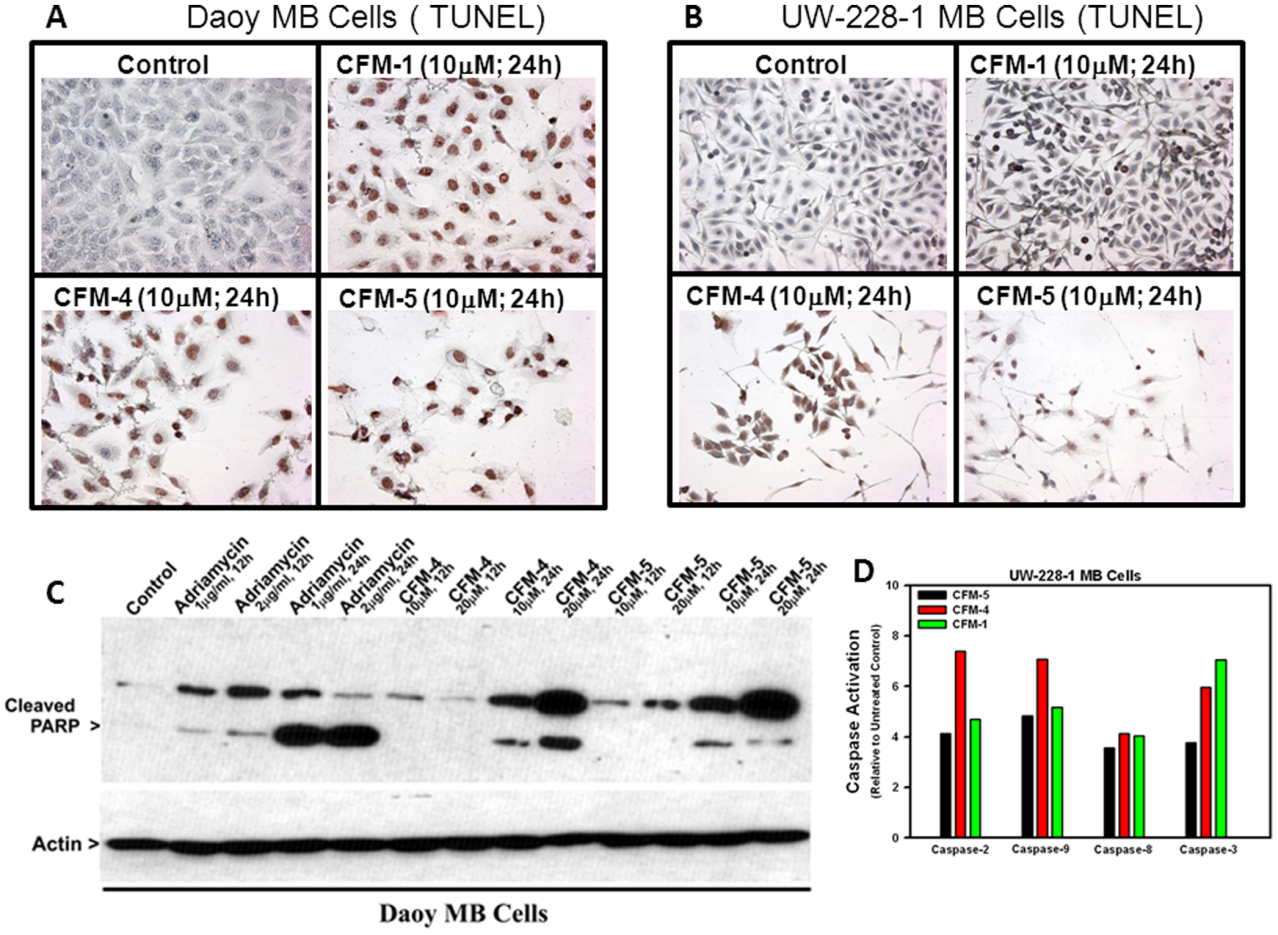
CFMs stimulate apoptosis in MB cells. (A, B) Indicated MB cells were either untreated (Control) or treated with different CFMs for indicated dose and time. Staining of the cells was performed using TUNEL assay as detailed in Methods. Dark brown staining represents fragmented cell nuclei. (C) MB cells were either untreated (Control) or treated with indicated agents for noted time and dose, and levels of PARP, cleaved PARP, and actin proteins were determined by Western blotting. (D) CFMs induce caspase activation in MB cells. Activities of the noted caspases were profiled as in Methods. Columns in histogram represent fold activities of caspases relative to the corresponding controls and are derived from means of two independent experiments.

We first tested the growth-inhibitory effects of CFMs on two human MB cell lines, Daoy and UW-228-1. CFM-1, 4, 5 inhibited the viability of both cell lines in a dose and time-dependent manner (Figure 1). In general, Daoy cells were more sensitive towards all the CFMs, showing ∼50% reduced viability in the presence of 20 µM CFM-1 (Figure 1A). Doses of 3 µM and 2 µM of CFM-4 and CFM-5, respectively, over a 24 h treatment elicited ∼50% reduced viability of Daoy cells (Figure 1B). The UW-228-1 cells were relatively less sensitive to all the CFMs since a 20 µM dose of CFM-1 caused ∼33% reduction in their viability (Figure 1A). Approximately 50% attenuation of the viability of UW-228-1 cells was noted when treated with a dose of 4 µM and 2 µM of CFM-4 and CFM-5, respectively, over a 24 h period (Figure 1B). In addition, since the Daoy and UW-228-1 MB cells were relatively more sensitive to CFM-4 or CFM-5, these cells were separately treated with various doses of CFM-4 and CFM-5 for a period of 24 h to determine the lowest effective dose for these agents. Although 500 nM dose of either compound failed to affect MB cells viability, a 1 µM dose of the either agent elicited a 20–40% loss in the viability of both the cell lines (Figure 1C, D). These data suggest that the viabilities of the MB cells are affected by the 1 µM or higher doses of each of the compound. We next investigated whether CFMs promoted apoptosis to inhibit MB cell growth. Given that a 12 h treatment of both the MB cells with 10 µM dose of CFM-1 resulted in a modest, 20–30-% loss of their viabilities, while a 12 h exposure to 10 µM dose of CFM-4 or CFM-5 suppressed viabilities of the MB cells by a 60–70%, we chose to utilize a 10 µM dose of each compound to determine induction of apoptosis and the underlying molecular mechanism(s). For immunocytochemical analyses, the MB cells were directly cultured in 8-well chamber slides and treated with CFMs as detailed in Methods. Treatment of the MB cells with CFM-1, 4, or 5 resulted in elevated number of TUNEL-positive cells (Figure 2A, B). Additional WB analysis revealed elevated cleavage of caspase-target poly(ADP-ribose) polymerase (PARP) following 24 h treatment of Daoy MB cells with the 10 and 20 µM dose of CFM-4 or CFM-5 (Figure 2C). Since the DNA-damage promoting chemotherapeutic Adriamycin (ADR) has been shown to induce PARP-1 cleavage and apoptosis in a number of cancer cells [25], our WB analysis also revealed cleavage of PARP in Daoy cells following treatment with ADR in a dose and time-dependent manner (Figure 2C). In light of our earlier studies indicating requirement of various caspase activities in transduction of CFM-4-dependent growth inhibitory signaling in human breast cancer cells [16], and the fact that CFMs promoted PARP cleavage in MB cells (Figure 2C), we next determined whether caspases were activated following exposure of the MB cells to CFMs. For this purpose, we profiled activation of caspases-2, -9, -8, and -3 in UW-228-1 cells following their treatments with CFM-1, -4, or -5 utilizing a fluorescence-based quantitative assay as noted in methods. As expected, treatments of MB cells with CFM-1, -4, or -5 resulted in activation of caspases-2, -9, -8, and -3 (Figure 2D). The data in figures 1 and 2 therefore suggest that CFMs are novel inhibitors of MB cell growth, and although the MB cells display varying levels of sensitivity towards these compounds, all the three CFMs significantly attenuate MB cell growth in part by stimulating apoptosis.

### Apoptosis Signaling by CFMs Involves Activation of Pro-apoptotic p38 SAPK/MAPK, and Elevated Expression of a Novel Transducer of Apoptosis CARP-1/CCAR

**Figure 3:**
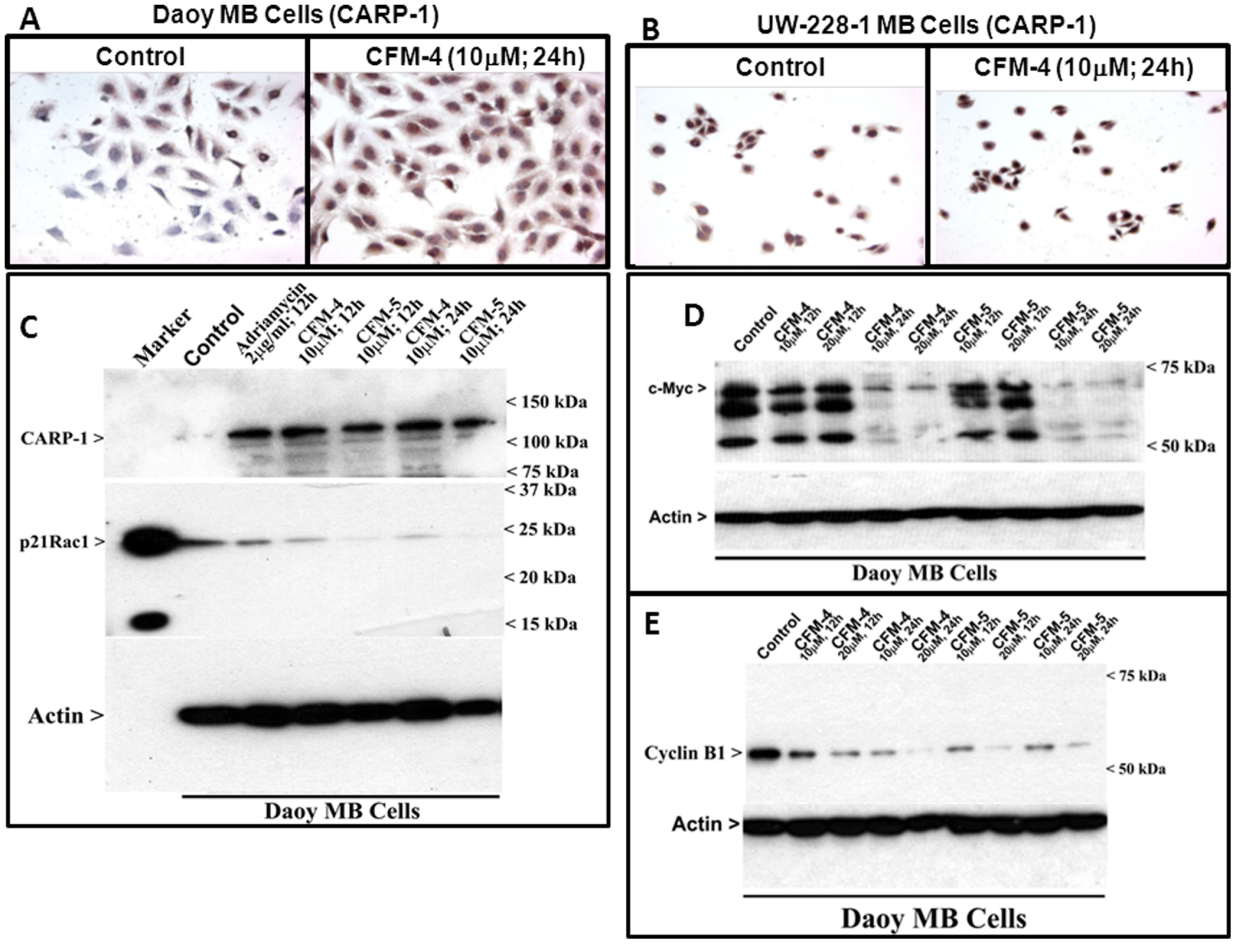
CFMs suppress MB survival and metastasis promoting genes while enhance expression of pro-apoptotic CARP-1. (A, B) Indicated MB cells were either untreated (Control) or treated with CFM-4 as in figure 2. Staining of the cells was performed using anti-CARP-1 (α2) antibody as detailed in Methods. Presence of CARP-1 is indicated by intense brown staining in the nuclei and cytosol of the treated cells. MB cells were either untreated (Control) or treated with different agents for indicated dose and time, and cell lysates were analyzed by western blotting for levels of CARP-1 (C), c-Myc (D), Cyclin B1 (E), and p21Rac1 (F) as in Methods.

We and others have previously reported identification and characterization of CARP-1/CCAR1 that is a novel and biphasic transducer of cell growth and apoptosis signaling. While CARP-1 was found to be a co-activator of nuclear, steroid/thyroid transcription factors, it was also demonstrated to be a key regulator of p53 function and a transducer of apoptosis signaling by DNA damaging agents such as ADR [14], [17]. Since both the MB cells displayed increased sensitivity to inhibition by CFM-4 and CFM-5, we next determined whether exposure of MB cells to these compounds caused increase in CARP-1 expression. In the first instance, the Daoy and UW-228-1 cells were either untreated or treated with CFM-4, and CARP-1 levels were analyzed by immuno-cytochemical staining utilizing anti-CARP-1 α2 antibodies as noted in Methods. Exposure to CFM-4 resulted in increased staining for CARP-1 in both the MB cells (Figure 3A, B). Consistent with elevated CARP-1 in CFM-4-treated Daoy cells, WB analysis of the lysates derived from ADR or CFM-4-treated Daoy cells revealed a robust increase in CARP-1 expression while CARP-1 presence was undetectable in the lysates from the untreated control cells (Figure 3C). Moreover, since CFM-5 inhibited growth of the Daoy cells (see figure 1) it also provoked an increase in CARP-1 levels (Figure 3C). The data in figures 3A-C demonstrate that ADR or CFM-4 enhance CARP-1 expression in the MB cells.

Our earlier studies have also indicated that in addition to regulating apoptosis signaling, CARP-1 regulated expression of a number of key cell cycle and cell growth regulatory molecules. In particular, knockdown of CARP-1 resulted in elevated levels of mitotic cyclin B1, cell growth and motility regulatory small GTP-binding protein p21Rac1, and oncogene c-myc [14]. Moreover, treatment of human breast cancer cells with CFM-4 resulted in reduced expression of cyclin B1 [16]. In light of these observations, we further investigated whether exposure of the MB cells to CFMs also results in loss of cyclin B1, p21Rac1, and c-myc proteins. As shown in figure 3 C, D, and E, CFM-4 or CFM-5 treatments suppressed expression of cyclin B1, p21Rac1, and c-myc proteins in Daoy MB cells.

**Figure 4:**
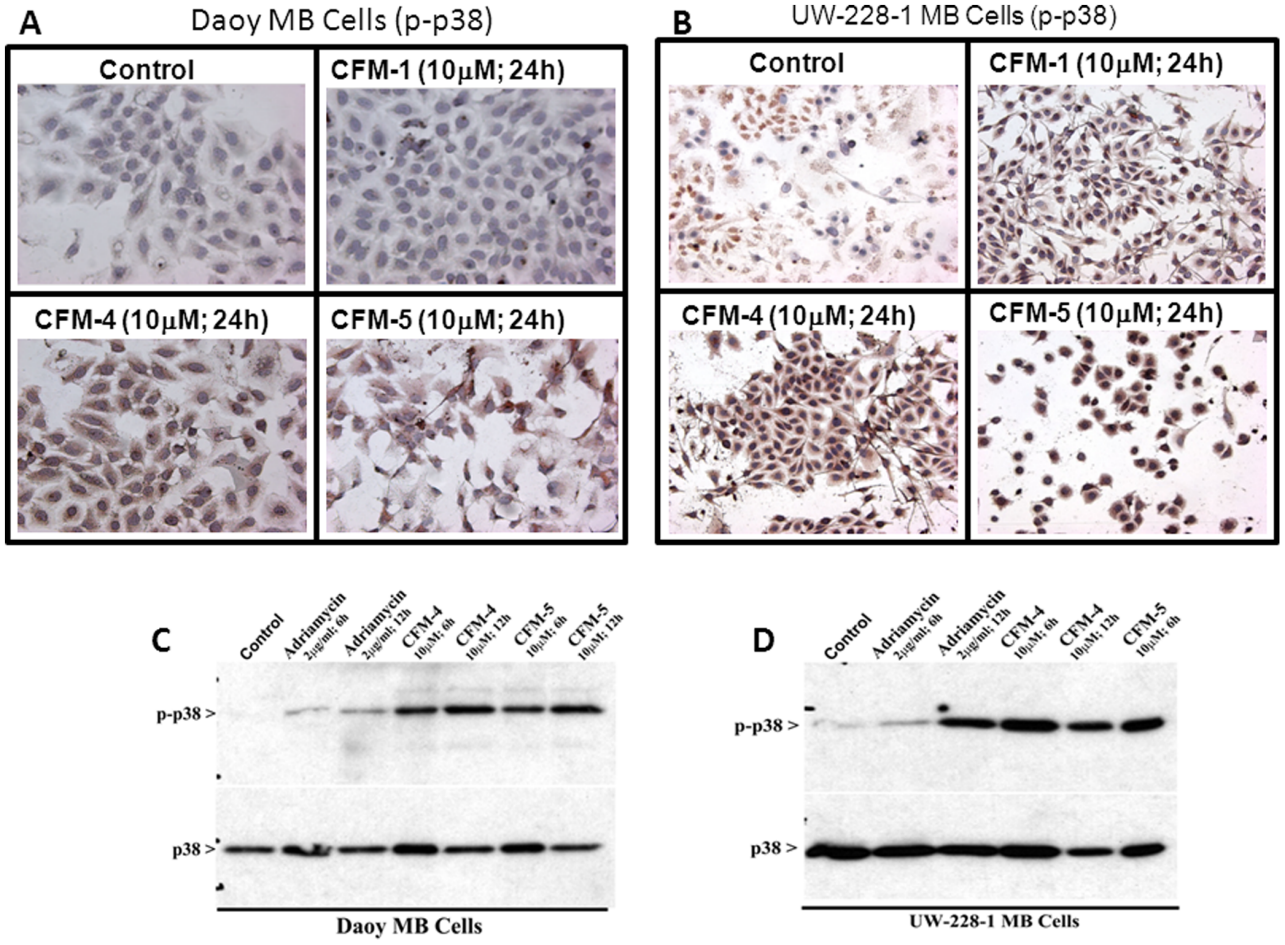
CFMs activate pro-apoptotic MAPK p38 in MB cells. (A, B) Indicated MB cells were either untreated (Control) or treated with noted agents as in figure 2. Staining of the cells was performed using anti-phospho-p38 antibody as detailed in Methods. Presence of p38 is indicated by intense brown staining in the nuclei and cytosol of the treated cells. (C, D) MB cells were either untreated (Control) or treated with indicated agents for noted time and dose, and levels of phosphorylated p38 (noted as p-p38), and total p38 proteins were determined by Western blotting.

We have previously observed that increased expression of CARP-1 stimulated apoptosis that involved activation of p38, while blockage of p38 activation abrogated CARP-1-mediated apoptosis [15]. In light of earlier findings demonstrating p38 activation during MB cell apoptosis [21], and together with our recent report describing identification and characterization of novel small molecule CFMs, in particular the CFM-4 that binds with CARP-1, increases CARP-1 levels, p38 activation and apoptosis [16], we speculated that CARP-1 expression and p38 activation are key events in MB cell apoptosis signal transduction in the presence of ADR or CFMs. To test this possibility, we determined whether and to what extent CFMs can stimulate p38 activation in the MB cells. Immuno-cytochemical and WB analyses were performed to determine CFM-mediated changes in p38 activation. Treatments with CFM-1, -4, or -5 resulted in elevated staining for phosphorylated (activated) p38 in Daoy (Figure 4A) and UW-228-1 (Figure 4B) MB cells when compared with their untreated counterparts. WB analysis of the cell lysates further corroborated a robust activation of p38 in ADR, CFM-4, or CFM-5-treated Daoy (Figure 4C) and UW-228-1 (Figure 4D) MB cells when compared with their untreated controls.

### CFMs Suppress MB Cell Growth in Part by Inducing Expression of Cell Growth and Survival Inhibitory Molecules

In an attempt to further elucidate molecular mechanisms of MB cell growth inhibition in the presence of CFMs, we first conducted a gene-array-based analysis. Daoy MB cells were either untreated or separately treated with three doses of CFM-4 as described in Methods. The RNAs from each group were hybridized with gene-array chips, and the data were analyzed to identify genes that had a significant 1.5-fold or higher altered expression following CFM-4 treatments. A select subset of these genes is indicated in table 1 (see Table S1 for complete list of CFM-4-regulated genes in Daoy MB cells). Interestingly, the array data revealed down-regulation of a vast majority of genes while a small number of gene were up-regulated. Among the genes that were down-regulated were cell growth and metastasis-promoting genes such as matrix metalloproteinase (MMP)7, MMP10, cyclin E1/E2, BUB1, transcription factor E2F2, E3 ubiquitin ligase SKP2, and small GTP binding proteins CDC42 and p21Rac1 (Table 1). Of note is the fact that CFM-4-dependent down-regulation of p21Rac1 in our array experiment is supportive of our WB data in figure 4E where both the CFM-4 and CFM-5 treatments attenuated levels of p21Rac1 in Daoy cells. The array data also revealed up-regulation of a number of cell growth inhibitory genes in the CFM-4-treated Daoy cells. In particular, expression of the tumor necrosis factor alpha inhibitory protein (TNFAIP)3-interacting proteins (TNIP) 1 and 2 [also known as A20-binding inhibitor of NF-κB activation (ABIN) 1 and 2], as well as the nuclear factor of kappa light polypeptide gene enhancer in B-cells inhibitor beta (NFκBIB, also known as IκBβ) were up-regulated suggesting that the CFM-4 treatment likely affected the NF-κB-dependent survival signaling in MB cells. In addition, CFM-4-treated MB cells had elevated expression of the receptor-interacting protein kinase (RIPK)4 and neurotrophin (NTF)3 genes (Table 1), that are known to transduce cell growth inhibitory functions in a context and signal-dependent manner.

**Table 1:** List of select CFM-4-regulated genes in MB cells.

SYMBOL	Name	REFSEQ	P.Value	Fold Change	Direction
EPCAM	epithelial cell adhesion molecule	NM_002354.2	6E-06	6.02320579	DOWN
MUC1	mucin 1, cell surface associated	NM_001018016.2	0.0034	3.4640361	DOWN
CLDN7	claudin 7	NM_001185022.1	0.0048	2.1547023	DOWN
MMP7	matrix metallopeptidase 7 (matrilysin, uterine)	NM_002423.3	0.0119	3.78874664	DOWN
WDR1	WD repeat domain 1	NM_005112.4	0.1832	1.68216064	UP
MMP10	matrix metallopeptidase 10 (stromelysin 2)	NM_002425.2	0.5942	2.06967364	DOWN
EGFR	epidermal growth factor receptor	NM_005228.3	0.6049	1.99298659	DOWN
CDC42	cell division cycle 42 (GTP binding protein, 25 kDa)	NM_001039802.1	0.6964	1.63084985	DOWN
DDR1	discoidin domain receptor tyrosine kinase 1	NM_001202521.1	0.7841	1.75939708	DOWN
CCNE1	cyclin E1	NM_001238.2	0.8052	2.10294057	DOWN
CLDN1	claudin 1	NM_001185056.1	0.8742	1.90736751	DOWN
TNIP2	TNFAIP3 interacting protein 2	NM_001161527.1	0.9213	1.51414051	UP
RAC1	ras-related C3 botulinum toxin substrate 1 (rho family, small GTP binding protein Rac1)	NM_006908.4	0.972	1.52048484	DOWN
CLDN3	claudin 3	NM_001306.3	0.9999	1.50957605	DOWN
DDX1	DEAD (Asp-Glu-Ala-Asp) box helicase 1	NM_004939.2	0.9999	1.66301833	DOWN
BUB1	budding uninhibited by benzimidazoles 1 homolog (yeast)	NM_004336.3	0.9999	1.50027115	DOWN
TOP2B	topoisomerase (DNA) II beta 180 kDa	NM_001068.2	0.9999	1.69726641	DOWN
NTF3	neurotrophin 3	NM_001102654.1	0.9999	1.64235481	UP
MAPK13	mitogen-activated protein kinase 13; SAPK-4	NM_002754.4	0.9999	1.91349836	DOWN
TNIP1	TNFAIP3 interacting protein 1	NM_001252385.1	0.9999	1.94721709	UP
SKP2	S-phase kinase-associated protein 2, E3 ubiquitin protein ligase	NM_001243120.1	0.9999	1.63679473	DOWN
TNFRSF9	tumor necrosis factor receptor superfamily, member 9	NM_001561.5	0.9999	1.97808931	UP
NFKBIB	nuclear factor of kappa light polypeptide gene enhancer in B-cells inhibitor, beta	NM_001243116.1	0.9999	1.74516006	UP
EFNB2	ephrin-B2	NM_004093.3	0.9999	1.61069326	DOWN
CCNE2	cyclin E2	NM_057749.2	0.9999	1.53626974	DOWN
E2F2	E2F transcription factor 2	NM_004091.3	0.9999	2.24474492	DOWN
RIPK4	receptor-interacting serine-threonine kinase 4	NM_020639.2	0.9999	1.67778842	UP

Nerve growth factor (NGF) and other members of the NTF family that function by activating receptor tyrosine kinases p75NTR and tropomyosin related kinases (trk) A, B, and C play important roles in survival, differentiation, and apoptosis of neurons [26]–[28]. In regard to the MBs, activation of TrkA and C receptors has been shown to stimulate apoptosis, while overexpression of TrkC receptor in childhood MBs is associated with favorable clinical outcome [21], [29], [30]. In light of the fact that NTF3 is a preferred ligand for TrkC receptor, and although low levels of 5.5-kb size alternate splice variant of TrkC mRNA were present in Daoy cells, no detectable levels of the 14-kb trkC, p75NTR, trkA, or trkB mRNAs as well as full-length p145^trkC^ receptor protein were found in these cells ([21] and refs within). Consequently, Daoy cells were not inhibited by NTF3 treatment, while stable expression of wild-type TrkC receptor in Daoy MB cells sensitized them to the inhibitory effects of NTF3 [21].

**Figure 5:**
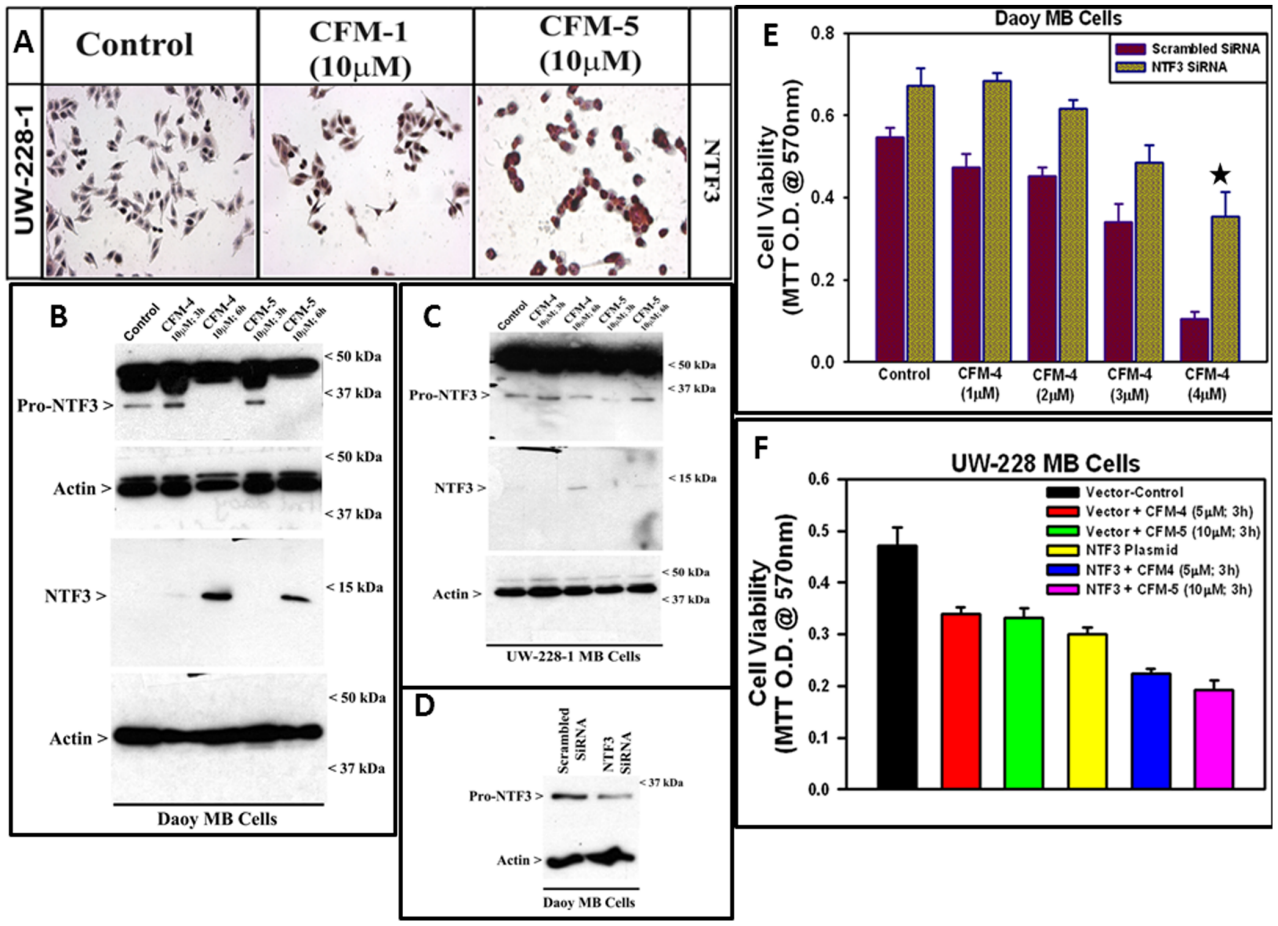
NTF3 expression regulates MB growth inhibitory signaling by CFMs. (A-C) CFM treatments result in elevated NTF3 levels in MB cells. In panel A, cells were either untreated (Control) or treated with noted doses of respective CFMs for 6 h. Staining of the cells was performed using anti-NTF3 antibody as detailed in Methods. Presence of NTF3 is indicated by intense brown staining in the nuclei and cytosol of the treated cells. In panels B and C, cells were either untreated (Control) or treated with CFMs for indicated dose and time, and cell lysates were analyzed by western blotting for levels of pro-NTF3, NTF3, and actin proteins as in Methods. (D, E) Knock-down of NTF3 interferes with MB cell growth inhibitory effects of CFM-4. MB cells were transfected with scrambled or NTF3 siRNAs as in Methods. In panel D, the cell lysates were analyzed for levels of pro-NTF3 and actin proteins by western blotting. In panel E, MB cells were transfected with siRNAs as in D, and were either untreated (Control) or treated with indicated doses of CFM-4 for 3 h. Determination of viable/live cells was carried out by MTT assay as in figure 1. The data in the histogram represents means of three independent experiments; bars, S.E. (∗, p = <0.03 relative to CFM-4-treated, scrambled siRNA-transfected cells). (F) NTF3 expression suppresses MB cell growth and enhances inhibitory effects of CFMs. MB cells were transiently transfected with vector or NTF3 expression plasmid as in Methods. Cells were either untreated (denoted as Vector-Control or NTF3 Plasmid) or treated with respective CFM for the noted dose and time. Determination of viable/live cells was carried out by MTT assay as in figure 1. The data in the histogram represent means of three independent experiments; bars, S.E.

Since gene-array experiment revealed upregulation of NTF3 in CFM-4-treated MB cells (see table 1), we next examined whether treatments of MB cells with CFM-4 or CFM-5 increased NTF3 levels. UW-228-1 cells were either untreated or treated with CFM-1 or CFM-5, and NTF3 levels were analyzed by immuno-cytochemical staining utilizing anti-NT3 mouse monoclonal antibody (clone 5A2) as noted in Methods. Exposure to CFM-5 resulted in increased staining for NTF3 in the UW-228-1 MB cells (Figure 5A). WB analysis further revealed a moderate increase in pro-NTF3 in the Daoy cells following their treatment with CFM-4 or 5 for 3 h period while a robust increase in mature NTF3 was noticed in these cells exposed to CFM-4 or 5 over a 6 h period (Figure 5B). A similar, albeit less robust, increase in the pro and mature NTF3 proteins was also noted in CFM-4 or CFM-5-treated UW-228-1 cells (Figure 5C). To determine the extent to which NTF3 is required in CFM-4-mediated growth suppression of MB cells, we conducted a siRNA-based knockdown of NTF3 in Daoy cells. In the first instance, the cells were transfected either with the scrambled siRNAs or NTF3-siRNAs as detailed in Methods, and the cell lysates analyzed by WB. Presence of NTF3-SiRNAs resulted in ∼50% loss of pro-NTF3 peptide when compared with its levels in scrambled siRNA-transfected counterparts (Figure 5D). We next transfected Daoy cells with scrambled or NTF3-siRNAs, followed by their treatments with vehicle DMSO (Control) or various doses of CFM-4 as in Methods. The viabilities of the siRNA-transfected, and control or CFM-4-treated cells were measured by the MTT assays essentially as in figure 1. MB cells transfected with scrambled siRNAs lost their viabilities in the presence of CFM-4 in a dose-dependent manner with a greater than 80% reduction in their viability by the 4 µM dose (Figure 5E). Knock-down of NTF3 however resulted in just ∼50% reduction in the number of viable cells in the presence of 4 µM CFM-4, thereby eliciting a statistically significant level of protection of MB cells from inhibitory effects of CFM-4. To further elucidate role of NTF3 in transducing MB-inhibitory effects of CFMs, we first generated and characterized stable, neomycin-resistant Daoy sublines that express NTF3 antisense as detailed in Methods. WB analysis revealed that over-expression of NTF3 antisense resulted in reduced levels of pro-NTF3 in a number of sublines when compared with their vector-transfected counterparts (Figure S1A). Two sublines, each expressing vector or NTF-3 antisense, were then independently treated with CFM-4 and CFM-5 for various time periods followed by determination of their viabilities as in Methods. Consistent with our data in figure 5E, stable knock-down of NTF3 interfered with the growth inhibitory effects of CFM-4 and CFM-5 (Figure S1B, C). These data therefore collectively suggest that MB cell growth inhibition by CFM-4 is regulated in part by NTF3-dependent signaling mechanisms.

In order to determine the mechanism by which increased expression of NTF3 by CFM-4 negatively regulated growth of the MB cells, we first clarified whether the Daoy and the UW-228-1 MB cells expressed any variants of TrkC, and to the extent to which CFMs can alter levels of these receptors to render cells sensitive to inhibition by NTF3. CFM-5-treated Daoy cells as well as CFM-1-treated UW-228-1 cells elicited elevated immuno-staining for TrkC protein (Figure S2A). In addition, cellular proteins derived from untreated (Control) and CFM-treated Daoy and UW-228-1 MB cells were subjected to WB analysis utilizing multiple anti-TrkC antibodies as described in Methods. The anti-TrkC mouse monoclonal antibody reacted with ∼100, 65, and 55 kDa size proteins in the Daoy cells while reacting only with ∼100 kDa size protein in the UW-228-1 cells (Figure S2B, C). Of note is the fact that although a modest increase in the 100 kDa TrkC protein was noted in CFM-4 as well as CFM-5-treated UW-228-1 cells, expression of the 100 kDa species was reduced while the levels of 65 and 55 kDa peptides was significantly increased in the CFM-4-treated Daoy cells (Figure S2B). Although the full-length TrkC receptor protein has been reported to be ∼145 kDa size, the available GenBank database information indicates presence of shorter sized, splice variants of the TrkC receptor ranging in sizes from 839 amino acids (NP_001012338.1), 825 amino acids (NP_002521.2), 817 amino acids (NP_001230030.1), to 612 amino acids (NP_001007157). Whether the 100 kDa species noted in the lysates of both the MB cells in figure S2B and S2C corresponds to any of the above splice variant of TrkC, and the extent increased expression of the 65 and 55 kDa species in CFM-4-treated Daoy cells is due in part to apoptotic signaling induced by CFM-4 remain to be clarified. Additional WB analysis of the lysates derived from UW-228-1 MB cells utilizing anti-TrkC rabbit monoclonal antibody (Cell Signaling Technology) also revealed presence of ∼100 kDa size peptide (Figure S2D). Taken together, our WB analyses in conjunction with multiple anti-TrkC antibodies suggest for the presence of a variant or a TrkC-like peptide(s) in the MB cells. We next addressed whether the TrkC-like peptide(s) contributed to NTF3-mediated MB cell growth suppression in the presence of CFM-4. MB cells were separately treated with CFM-4, affinity-purified pro-NTF3, affinity-purified mature NTF3, or a combination of CFM-4 and pro/mature NTF3, and cell viability was measured by MTT assay as in figure 1. Although treatment with a 5 and 50 ng/ml dose of pro-NTF3 elicited a modest (∼20%) growth inhibition of the UW-228-1 MB cells, neither mature NTF3 nor a combination of CFM-4 and NTF3 resulted in a greater growth inhibition when compared with that noted with the CFM-4-treated cells (Figure S3A, S3B). These data collectively suggest that although these MB cells express TrkC-like peptides, the elevated levels of NTF3 following treatments with CFM-4 or CFM-5 are not likely to be involved in extrinsic, receptor-dependent growth inhibitory signaling.

We next tested the possibility that elevated levels of intracellular NTF3 in the presence of CFM-4 or CFM-5 contribute to MB cell growth inhibition in the absence of functional TrkC. For this purpose, a plasmid encoding variant 1 of the human pro-NTF3 protein was transfected in the MB cells. Transfection of the NTF3 expression plasmid however resulted in elevated levels of NTF3 in the UW-228-1 cells (Figure S3C). The vector or NTF3 plasmid transfected UW-228-1 cells were then either untreated or treated with CFM-4 or CFM-5, followed by determination of live, viable cells essentially as in figure 1. As shown in figure 5F, expression of NTF3 significantly attenuated viabilities of the UW-228-1 MB cells when compared with their vector-transfected controls. Moreover, the extent of the loss of viability of UW-228-1 cells following NTF3 expression was similar to that noted for the CFM-4 or CFM-5-treated, vector-transfected cells (Figure 5F). Importantly, treatments of NTF3-expressing UW-228-1 cells with CFM-4 or CFM-5 resulted in greater reductions in their viability when compared to their CFM-4 or CFM-5-treated, or NTF3-transfected counterparts (Figure 5F). Although the lipid-based transfection reagent lipofectamine 2000 induced greater cell death in the transfected Daoy cells when compared with the UW-228-1 MB cells, transfection of the NTF3 plasmid nonetheless elicited greater loss of viability of the Daoy cells when compared with their vector transfected controls (figure S3D). The data in figures 5E and F suggest that, in the absence of functional TrkC, elevated levels of intracellular NTF3 following treatment of MB cells with CFM-4 or CFM-5 contribute to the pleiotropic growth inhibitory effects of CFMs. The precise nature of this signaling and the mechanism(s) by which elevated NTF3 suppresses MB cell growth in the presence of CFMs remain to be clarified. Nonetheless, the property of the CFMs to suppress MB growth in part by stimulating NTF3 production could be useful in suppression of the TrkC-positive MBs and together with our current studies with the TrkC-negative MB cells, would underscore a broader potential for this signaling in effectively inhibiting a diverse set of MBs.

### CFM-4 Activates JNK MAPKs While Regulating the Canonical NF-κB Signaling in a Biphasic Manner

**Figure 6:**
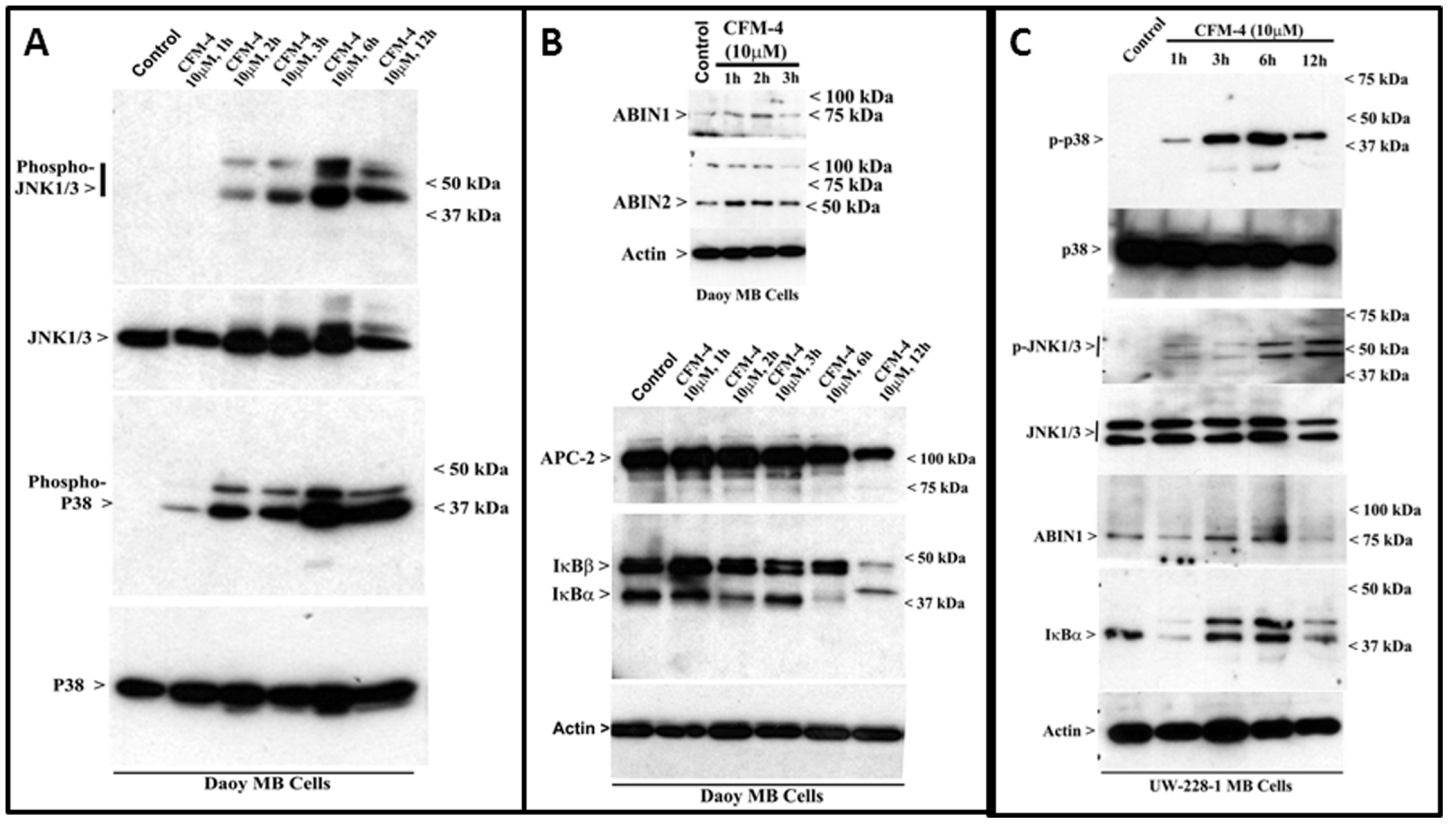
CFM-4 activates pro-apoptotic JNK while causes a biphasic regulation of canonical NF-κB signaling. Cells were either untreated (Control) or treated with CFM-4 for indicated dose and time, and cell lysates were analyzed by western blotting for levels of pro-apoptotic, phospho-JNK, phosphorylated p38, total JNK1/3, total p38 (A, C), ABIN1, ABIN2, APC-2, IκBα, IκBβ and actin (B, C) proteins as indicated in Methods.

As both the CFM-4 and CFM-5 activated pro-apoptotic p38 MAPK/SAPK (figure 4), we further determined whether the c-Jun N-terminal Kinase (JNK), another key member of the MAPK/SAPK family was also activated by our compounds. Both the Daoy and UW-228-1 MB cells were treated with a 10 µM dose of CFM-4 for different time periods. The cell lysates were analyzed by WB and activation of JNK1/3 was compared with the activation levels of p38. The p38 activation was noticeable in both the MB cell lines within 1 h of CFM-4 treatment while robust p38 activation occurred at the 2 h and 3 h of CFM-4 treatment of Daoy and UW-228-1 cells, respectively (Figure 6A, C). CFM-4 treatment also elicited a modest activation of JNK at the 1–3 h time points in both cell lines. A robust activation of JNK however was noticeable at the 6 h and later periods of CFM-4 exposure (Figure 6A, C). These data suggest that p38 activation likely precedes JNK activation, and taken together indicate that activation of both the pro-apoptotic MAPKs contribute to the MB cell growth inhibitory effects of CFM-4.

In light of our gene-array data that revealed elevated levels of a number of key molecules that are well known to inhibit canonical NF-κB signaling (Table 1), we further clarified the extent to which CFM-4 treatments can upregulate levels of ABIN1, ABIN2, IκBα and IκBβ proteins in MB cells. MB cells were treated with CFM-4 essentially as is figure 6A and C, and expression/levels of NF-κB-inhibitory proteins were determined by WB. A moderate increase in levels of ABIN1 and 2 proteins was noticeable within 1 h of CFM-4 treatment of Daoy cells (Figure 6B), while only ABIN1 levels increased after 3 h and 6 h of CFM-4-treated UW-228-1 cells (Figure 6C). Interestingly, although a moderate increase in IκBβ was noted within 1 h of CFM-4 treatment, no significant increase in IκBα levels was evident in the Daoy cells. In the case of UW-228-1 cells however CFM-4 exposure resulted in initial decline in IκBα levels followed by re-expression of IκBα at 3 h and 6 h treatment periods (Figure 6C). These data would collectively suggest for a biphasic regulation of NF-κB signaling by CFM-4 in the MB cells. Since ABIN1 and 2 proteins negatively regulate NF-κB function in part by interfering with activation of its upstream kinase IKK [31], [32], it is likely that a modest increase of ABIN1 and 2 proteins in the presence of CFM-4 likely inhibits canonical NF-κB signaling as an early response while prolonged exposure results in degradation of/reduced expression of IκBα and/or IκBβ proteins in both the MB cells. It remains to be clarified whether loss of IκBα and/or IκBβ following prolonged exposure to CFM-4 results in NF-κB activation, and to what extent activated NF-κB functions to stimulate apoptosis or promotes cell survival to overcome CFM-4-mediated stress.

### CFM-4 Blocks Migration, Colony Formation, and Invasion of MB Cells

**Figure 7:**
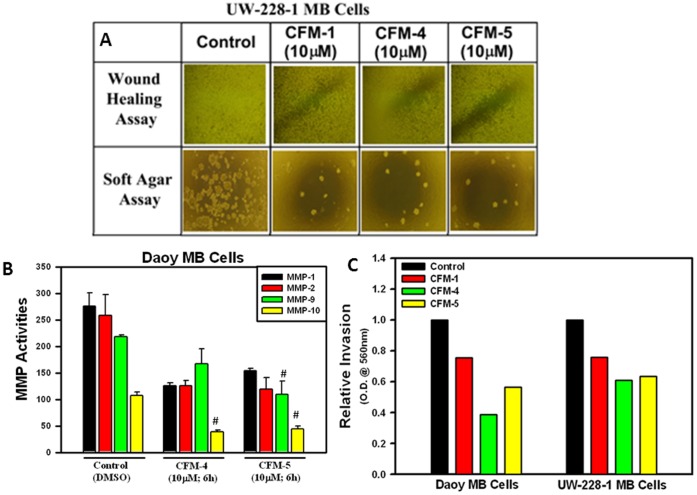
CFMs inhibit MB Cell Growth in Soft Agar, invasion and MMP activities. (A) MB cells were either untreated (Control) or treated with indicated dose of respective CFMs, and were subjected to the subjected to the scratch assays (indicated as wound healing assay; upper panel) or soft-agar assay (lower panel). The cells in the scratch assay or the colonies of cells in soft-agar were photographed as described in Methods. Representative photomicrographs of untreated and CFM-treated UW-228-1 cells are shown. (B) The indicated MB cells were either untreated [Control (DMSO)] or treated with CFM-4 or CFM-5 for noted dose and time. Cell lysates were analyzed for activities of various MMPs as detailed in Methods. The data in the histogram represents means of the activities of the noted MMPs from three independent experiments; bars, S.E. (C) The MB cells were separately seeded in chambers with matrigel-coated membranes, and treated with buffer (Control) or with 10 µM dose of respective CFMs as noted in Methods. Live cells migrating across the matrigel-coated membranes were dissociated, and quantitated by an MTT-based assay. The columns in histogram represent MTT OD of the CFM-treated MB cells relative to untreated controls. (#, p =  <0.05 relative to Control (DMSO)-treated cells).

To investigate whether CFMs inhibit biological properties of migration, invasion and colony formation by the MB cells, we performed wound-healing, soft-agar, and matrigel-invasion assays, respectively, as detailed in Methods. Presence of CFM-1, -4, or -5 prevented the UW228-1 cells from growing in the areas of wound created by a scratch, and also caused a greatly reduced number and size of their colonies in soft agar when compared with their respective, untreated controls (Figure 7A). The fact that CFM-4 treatment of Daoy cells resulted in a 2-fold down-regulation of MMP10 (Table 1), and that the activities of MMPs were found to be associated with glioblastoma and MB cell invasiveness [33]–[35], we determined whether and to what extent exposure to CFMs resulted in reduced activities of various MMPs. For this purpose we conducted an antibody-based array analysis to determine activation status of various MMPs in control (untreated) versus treated Daoy MB cells as indicated in Methods. These data revealed that exposure of Daoy cells to CFM-4 or CFM-5 resulted in attenuation of MMP-1, -2, -9, and -10 activities (Figure 7B). SiRNA-mediated specific knock-down of either membrane type 1-MMP (MT1-MMP; MMP-14; a direct activator of MMP-2) or MMP-9 was recently found to reduce capacity of the Daoy cells to generate neurospheres while concomitantly abrogating their invasive capacity [36]. Whether CFM-dependent attenuation of MMP activities interfered with invasive properties of the MB cells was determined next by testing the extent to which CFMs blocked the ability of MB cells to invade through the matrigel-coated membranes. As expected, treatment of MB cells with CFM-1, -4, or -5 resulted in a significantly reduced number of MB cells that were able to migrate across the matrigel-coated membranes (Figure 7C). Taken together, these data show that CFMs, in particular CFM-4 and -5, have the ability to interfere with MB cell invasion and metastasis-inducing pathways.

## Discussion

The proof-of-concept studies described here highlight MB cell growth inhibitory properties of CFMs, a novel class of compounds. Consistent with the ability of CFMs to inhibit growth of the breast and other cancer cells [16], we found that CFMs stimulate apoptosis signaling while suppressing molecules/pathways regulating MB cell cycle and cell growth. Anti-cancer molecules/compounds that simultaneously block cancer cell growth and survival pathways while activating apoptosis are likely to be superior in effectively suppressing cancer. Consistent with this expectation, our current studies demonstrate that CFM-4 suppressed MB cells growth by activating caspases and inducing apoptosis signaling as well as by diminishing the levels of cell cycle regulatory protein cyclin B1. In addition to stimulating CARP-1 expression, activation of pro-apoptotic SAPKs (p38 and JNK), and various caspases, our current studies demonstrate for the first time that MB cell growth inhibitory signaling activated by CFMs involved up-regulation of NTF3 and biphasic regulation of NF-κB signaling, while key transducers of invasion and metastasis pathways were down-regulated.

A number of published studies have described pleiotropic effects of NGF and various other neurotrophins in neuronal cell types that range from regulation of survival, proliferation and cell death [37], [38]. Moreover presence of neurotrophin and their receptors has also been demonstrated in primary MB specimens while presence of TrkC receptors has been shown to correlate with a better response to therapy [6], [39]. Growth inhibitory effects of neurotrophins, particularly NGF and NTF3, have been further highlighted in the Trk-negative cells that were engineered to overexpress TrkA or TrkC receptors [21], [28], [30]. In addition, Pro-neurotrophins have also been shown to promote neuronal cell death in the presence or absence of Trk receptors. In the cells that lack Trk receptors, the pro-apoptotic signaling by pro-neurotrophins often involve p75NTR and/or sortilin receptors [40]. Our current data in table 1 and figure 5 indicate that levels of both the pro and mature NTF3 were elevated in MB cells following their treatments with CFMs. Since MB cell lines generally do not express functional NTR and Trk receptors [30], and although our WB analysis in conjunction with multiple anti-TrkC antibodies showed presence of TrkC-like peptides in the MB cells, their treatments with purified pro-NTF3 or mature NTF3 not only failed to elicit growth inhibition but also did not enhance inhibitory effects of CFMs (see figures S2 and S3). Interestingly however knock-down of NTF3 interfered with CFM-4-dependent inhibition of Daoy cells (Figure 5) suggesting that intracellular levels of NTF3 were likely involved in transducing MB growth inhibitory effects of CFMs. Indeed, transient overexpression of NTF3 not only reduced the number of viable MB cells when compared with their vector expressing counterparts (Figures 5 and S3D), transfection of NTF3 expression plasmid further reduced viabilities of the MB cells in the presence of CFM-4 or CFM-5. Thus CFMs or their class of molecules could potentially be useful inhibitors of MBs independent of their NTR and/or Trk expression.

The neuronal apoptosis signaling by the NTFs in the NTR-expressing cells has been shown to involve activation of the cJun N-terminal kinase (JNK) while NTR proteolysis and subsequent apoptosis was abrogated in the sympathetic neurons of jnk3−/− mice, indicating a requirement for JNK signaling in neuronal apoptosis [41]–[43]. Although CFM-4 has been demonstrated to activate pro-apoptotic p38SAPK in breast cancer cells [16], our current studies indicate that in addition to p38SAPK, CFM-4 also activates JNK in MB cells (see figures 4 and 6). It however appears that a robust activation of p38 occurs earlier than robust JNK activation in both the MB cells (Figure 6). These data underscore pleiotropic nature of apoptosis signaling by CFMs, and suggest that combined activation of p38 and JNK likely amplify the apoptosis signals in the MB cells independent of the involvement of the cell surface NTR and Trk receptors. Moreover, JNK activation has also been implicated in processing of NTR in the neuronal cells [40]. Since CFM-4 and CFM-5 also enhanced production of mature NTF3 in both the MB cells (Figure 5), to the extent JNK activation also contributed to CFM-dependent processing of pro-NTF3 independent of NTR or Trk receptors in these MB cells remains to be clarified.

The NF-κB family of proteins and their signaling is well known to play a crucial role in organismal physiology and pathologies such as chronic inflammation and cancer. NF-κB signaling however also antagonizes apoptosis induced by TNF-α due in part to suppression of JNK activation [44]. Consistent with these findings, activation of pro-apoptotic MAPKs (p38 or JNK) serves to attenuate NF-κB activation in different stress-induced apoptotic contexts [45], [46]. Our data in figure 6 show that CFM-4 exposure over a periods of upto 6 hours results in a slight to modest decline in levels of NF-κB inhibitory IκBα and/or IκBβ proteins. Together with our data indicating a modest increase in NF-κB signaling inhibitory proteins ABIN1 and 2 [31], [32] following treatments of MB cells with CFM-4 (figure 6), it is likely that growth inhibitory signaling by CFM-4 impacts the canonical, IKK-dependent NF-κB signaling pathway [47]. Interestingly prolonged exposure to CFM-4 resulted in diminished expression of the NF-κB inhibitory IκBα and/or IκBβ proteins (figure 6). Whether this CFM-4-dependent loss of IκBs is due to degradation of cellular proteins as a consequence of apoptosis induction or a signal for NF-κB activation to antagonize or promote apoptosis is unclear. The activation of NF-κB following prolonged exposure to CFM-4 would nevertheless be consistent with the pro-survival functions of NF-κB signaling to antagonize/overcome the stress of CFM-4 exposure. On the other hand, given that a number of recent studies have revealed a pro-apoptotic role for NF-κB signaling [48]–[50], it is also likely that NF-κB activation following prolonged exposure to CFM-4 serves to potentiate/support apoptosis in the MB cells. Further studies will be necessary to clarify the precise role of NF-κB signaling in pleiotropic cell growth inhibitory effects of CFM-4.

Last but not least, the gene array data together with our observations in figure 7 suggest that CFMs impact the biological properties of migration and invasion by MB cells. Since small GTP-binding protein p21Rac1 is well known to regulate processes of cellular motility, loss of p21Rac1 expression following treatment of MB cells with CFMs would be consistent with their inhibition of MB cell motility and migration. In addition, expression/activation of various MMPs was also impacted by CFMs as indicated by our data in figure 7 and table 1. Given that MMPs are downstream targets of NF-κB signaling and their expression and activation contribute to the NF-κB-dependent survival and metastatic spread of the cancer cells, our findings would further highlight anti-invasion and metastasis-inhibitory properties of CFMs. The precise mechanisms by which CFMs impact p21Rac1 and MMP expression are the subjects of our on-going studies.

In summary, the data presented here convincingly demonstrate that CFMs activate multiple cell growth inhibitory and apoptosis pathways to suppress MB cell growth, survival and metastasis processes, and underscore their potential as novel class of anti-MB agents.

## Supporting Information

Figure S1 **Stable knock-down of NTF3 interferes with inhibitory effects of CFMs.** (A) MB cells were transfected with vector or NTF3-AS clone 1 plasmids, and stable, neomycin-resistant sublines were isolated as in Methods. Cell lysates were analyzed by western blotting for levels of pro-NTF3 and actin proteins as in figure 6. (B, C). The indicated sublines were either untreated (Control) or treated with noted doses of respective CFM for various times. Determination of viable/live cells was carried out by MTT assay as in figure 1. The data in the histograms represent means of three independent experiments; bars, S.E.
(TIF)

Figure S2 **Expression of TrkC-like peptides in MB cells.** (A) MB cells were either untreated (Control) or treated with noted doses of respective CFMs for 6 h. Staining of the cells was performed using anti-TrkC antibody (Santa Cruz, Biotech) as detailed in Methods. In panels B-D, cells were either untreated (Control) or treated with CFMs for indicated dose and time, and cell lysates were analyzed by western blotting for levels of TrkC-like peptides and actin proteins as in Methods. Of note is the fact that for western blots of panels B and C anti-TrkC mouse monoclonal antibody (Life Span Biosciences) was utilized while the membrane in panel D was probed with anti-TrkC antibody (Cell Signaling).
(TIF)

Figure S3 **Treatments of MB cells with purified, mature NTF3 (A) or purified, pro-NTF3 (B) does not inhibit MB cell growth.** MB cells were either untreated (denoted as -), pre-treated with noted doses of NTF3 or pro-NTF3 for 12 h, in the absence or presence of respective CFMs as indicated. Determination of viable/live cells was carried out by MTT assay as in figure 1. The data in the histograms represent means of three independent experiments; bars, S.E. Expression (transfection) of NTF3 plasmid results in increased levels of pro-NTF3 (C) and decreased cell viability (D). For panel C, cells were transfected with vector or NTF3 plasmid and cell lysates were analyzed by western blotting for levels of Pro-NTF3 and actin proteins as in Methods. For panel D, determination of viable/live cells was carried out by MTT assay utilizing lysates of vector or the NTF3 plasmid-transfected MB cells essentially as in figure 1. The data in the histogram represents means of three independent experiments; bars, S.E.
(TIF)

Table S1 **List of CFM-4-regulated genes in Daoy MB cells.**
(XLSX)
