# Supplementary figures and images for: CARP-1 Functional Mimetics: A Novel Class of Small Molecule Inhibitors of Medulloblastoma Cell Growth
Source: PLoS One. 2013 Jun 24;8(6):e66733. doi: 10.1371/journal.pone.0066733 (PMC3691183; doi:10.1371/journal.pone.0066733)

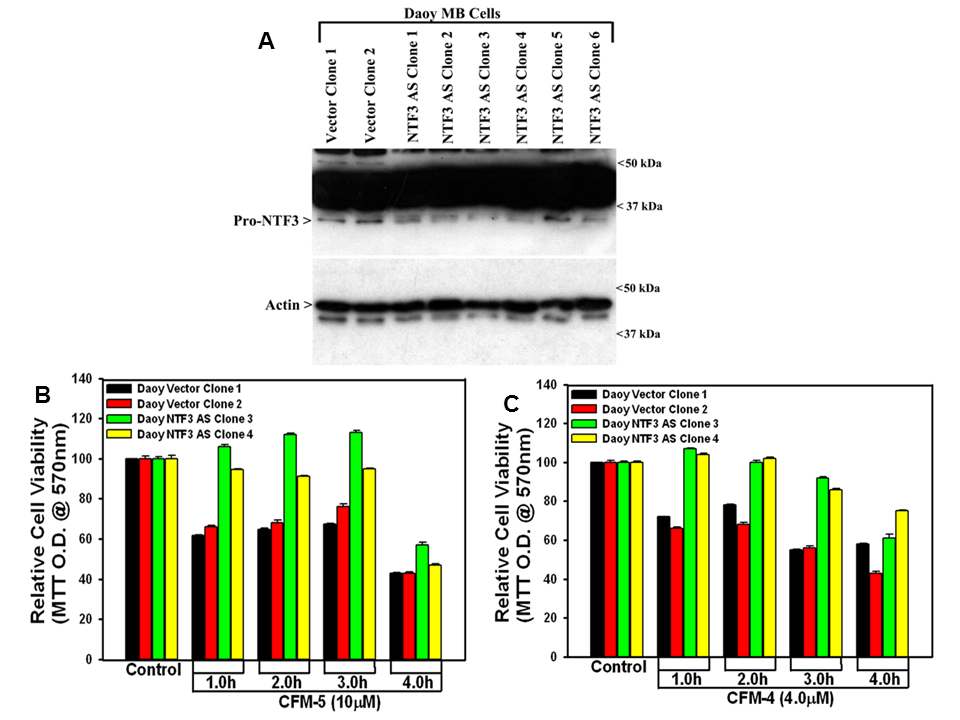

Supplement: Figure S1 — Stable knock-down of NTF3 interferes with inhibitory effects of CFMs. (A) MB cells were transfected with vector or NTF3-AS clone 1 plasmids, and stable, neomycin-resistant sublines were isolated as in Methods. Cell lysates were analyzed by western blotting for levels of pro-NTF3 and actin proteins as in figure 6. (B, C). The indicated sublines were either untreated (Control) or treated with noted doses of respective CFM for various times. Determination of viable/live cells was carried out by MTT assay as in figure 1. The data in the histograms represent means of three independent experiments; bars, S.E. (TIF) [file pone.0066733.s001.tif]

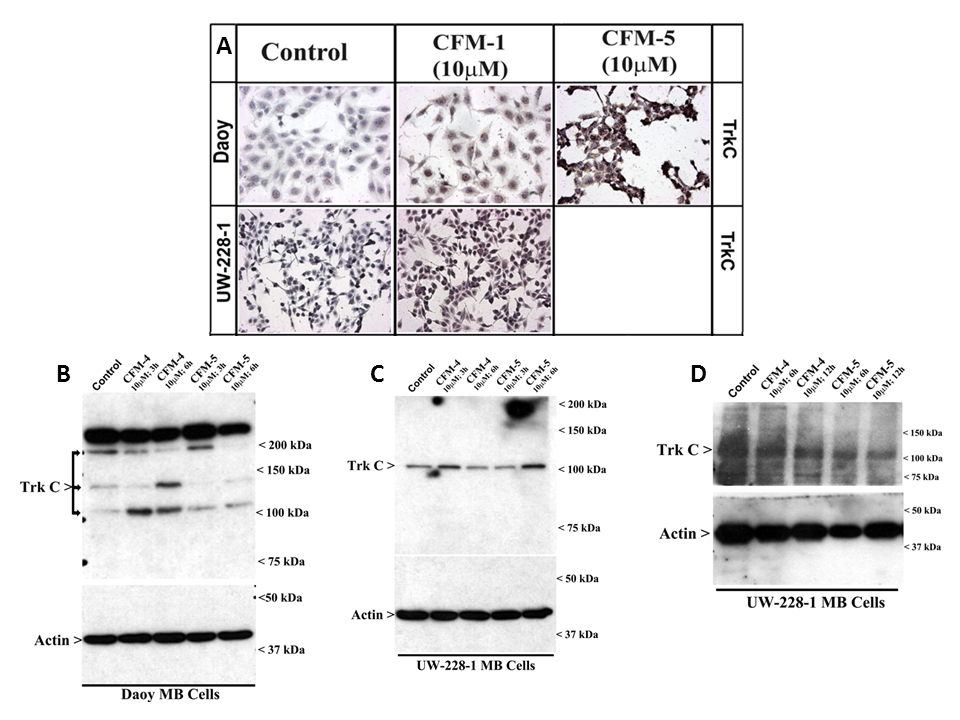

Supplement: Figure S2 — Expression of TrkC-like peptides in MB cells. (A) MB cells were either untreated (Control) or treated with noted doses of respective CFMs for 6 h. Staining of the cells was performed using anti-TrkC antibody (Santa Cruz, Biotech) as detailed in Methods. In panels B-D, cells were either untreated (Control) or treated with CFMs for indicated dose and time, and cell lysates were analyzed by western blotting for levels of TrkC-like peptides and actin proteins as in Methods. Of note is the fact that for western blots of panels B and C anti-TrkC mouse monoclonal antibody (Life Span Biosciences) was utilized while the membrane in panel D was probed with anti-TrkC antibody (Cell Signaling). (TIF) [file pone.0066733.s002.tif]

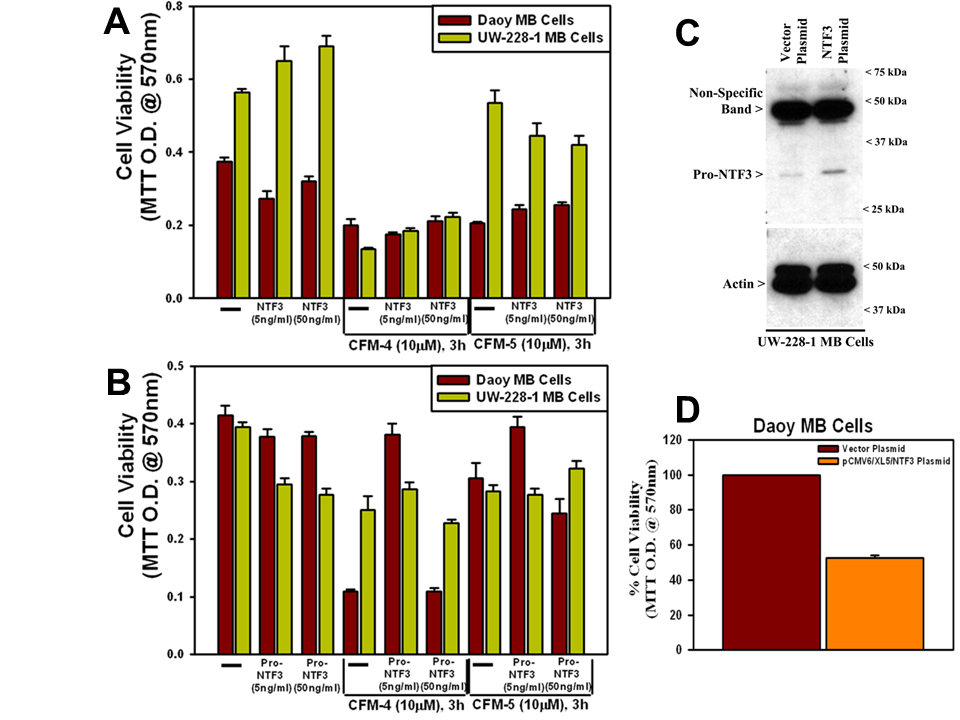

Supplement: Figure S3 — Treatments of MB cells with purified, mature NTF3 (A) or purified, pro-NTF3 (B) does not inhibit MB cell growth. MB cells were either untreated (denoted as -), pre-treated with noted doses of NTF3 or pro-NTF3 for 12 h, in the absence or presence of respective CFMs as indicated. Determination of viable/live cells was carried out by MTT assay as in figure 1. The data in the histograms represent means of three independent experiments; bars, S.E. Expression (transfection) of NTF3 plasmid results in increased levels of pro-NTF3 (C) and decreased cell viability (D). For panel C, cells were transfected with vector or NTF3 plasmid and cell lysates were analyzed by western blotting for levels of Pro-NTF3 and actin proteins as in Methods. For panel D, determination of viable/live cells was carried out by MTT assay utilizing lysates of vector or the NTF3 plasmid-transfected MB cells essentially as in figure 1. The data in the histogram represents means of three independent experiments; bars, S.E. (TIF) [file pone.0066733.s003.tif]
